# Supplementary figures and images for: Mutational scan of self-cleavage by HIV-1 protease provides new views of a conformationally dynamic mechanism
Source: J Virol. 2026 Mar 31;100(4):e02229-25. doi: 10.1128/jvi.02229-25 (PMC13098228; doi:10.1128/jvi.02229-25)

Figure S1

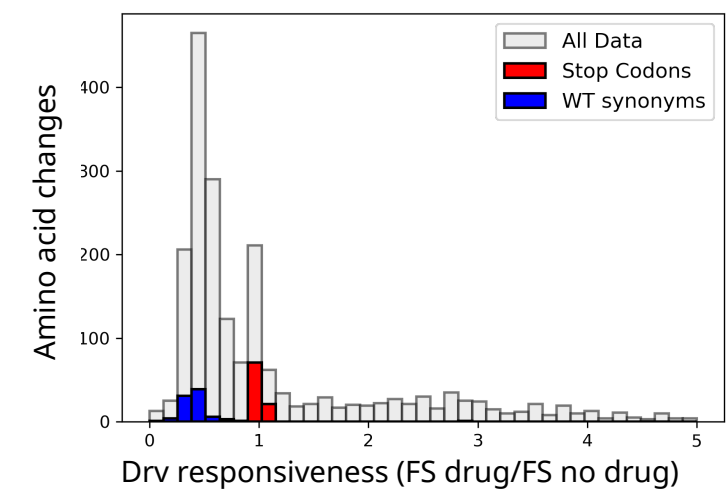

Figure S1. Distribution of DRV responsiveness.

Supplement: Figure S1 — Distribution of DRV responsiveness. [file jvi.02229-25-s0001.pdf]
